# Alternatives to antibiotics in pig production: looking through the lens of immunophysiology

**DOI:** 10.1007/s44154-023-00134-w

**Published:** 2024-01-02

**Authors:** Hao-Yu Liu, Chuyang Zhu, Miaonan Zhu, Long Yuan, Shicheng Li, Fang Gu, Ping Hu, Shihao Chen, Demin Cai

**Affiliations:** 1 College of Animal Science and Technology, Yangzhou University, Yangzhou, 225009 PR China; 2 Joint International Research Laboratory of Agricultural & Agri-Product Safety, The Ministry of Education of China, Yangzhou University, Yangzhou, China

**Keywords:** Antibiotics, Gut-associated lymphoid tissues, Probiotics, Prebiotics, Phytobiotics, The Hygiene Hypothesis

## Abstract

In the livestock production system, the evolution of porcine gut microecology is consistent with the idea of “The Hygiene Hypothesis” in humans. *I.e.*, improved hygiene conditions, reduced exposure to environmental microorganisms in early life, and frequent use of antimicrobial drugs drive immune dysregulation. Meanwhile, the overuse of antibiotics as feed additives for infectious disease prevention and animal growth induces antimicrobial resistance genes in pathogens and spreads related environmental pollutants. It justifies our attempt to review alternatives to antibiotics that can support optimal growth and improve the immunophysiological state of pigs. In the current review, we first described porcine mucosal immunity, followed by discussions of gut microbiota dynamics during the critical weaning period and the impacts brought by antibiotics usage. Evidence of in-feed additives with immuno-modulatory properties highlighting probiotics, prebiotics, and phytobiotics and their cellular and molecular networking are summarized and reviewed. It may provide insights into the immune regulatory mechanisms of antibiotic alternatives and open new avenues for health management in pig production.

## Introduction

The routine usage of antibiotics to maintain animal health and productivity has been a hallmark of modern animal husbandry. In pig production, antimicrobials are not only used as therapeutics against pathogenic bacteria; but also as prophylaxis to prevent infection; and as antibiotic growth promoters (AGPs) to improve production efficiency (Van Boeckel et al. 2015; Waluszewski et al. 2021), where the average growth rate of pigs was improved between 4-8% (Li 2017; Luecke et al. 1951). However, the overuse of antibiotics in livestock has led to the dissemination of antimicrobial resistance (AMR) genes into pathogenic bacteria, causing drug-resistant infections in humans (Mestrovic et al. 2022; Van Boeckel et al. 2015), in addition to impairing the host intestinal development, metabolism homeostasis, and even showing transgenerational effects (Cox et al. 2014; de Greeff et al., 2020; Zarrinpar et al. 2018). Concerns arise as low-dose of repeated antibiotic administration in farming could promote AMR by selection pressure; contaminate the environment with antibiotic-resistant bacteria, transferring AMR horizontally; and produce animal products with antibiotic residuals, causing *de novo* AMR genes and bacteria (Aslam et al. 2021; Rahman et al. 2022). A cardinal study established a comprehensive pig gut microbiome gene reference catalogue revealed that the highest prevalent AMR genes are resistant to tetracycline, macrolide, bacitracin, cephalosporin, and streptogramin B (Xiao et al. 2016). While metagenomic data analysis of pig and human intestinal samples uncovered a shared core resitome of 27 AMR genes (Wang et al. 2021a). In 2016, the emergence of plasmid-mediated colistin resistance gene *MCR-1* in *Enterobacteriaceae* from pigs and human samples was identified, where colistin is considered the last resort of antibiotics for treating drug-resistant bacterial infections (Liu et al. 2016).

In this context, a global effort has been made to reduce antibiotic usage in the livestock sector. The earliest in-feed AGPs ban was legislated in Sweden and Denmark and put into effect in Europe by 2006 (Aidara-Kane et al. 2018; Waluszewski et al. 2021). Whereas China, the largest producer of pigs and the largest consumer of veterinary antimicrobials, unveiled its National Action Plan to combat AMR in 2016 and has now officially entered the era of “no antibiotics in feed” (China 2016; Tian et al. 2021). However, the ban on in-feed antibiotic usage has unintended impacts on pig production, such as increased morbidity and mortality of infectious diseases and higher associated economic losses. In addition, despite the variation in classes and the route of application, antibiotics are still massively used in livestock (Cuong et al. 2018; Lekagul et al. 2019; Waluszewski et al. 2021). The global antibiotic usage for domestic animals was estimated at 68,535–193,052 tons in 2020 and was projected to increase by 8% in 2030 (Mulchandani et al. 2023). Therefore, finding antibiotic alternatives to manage animal health and production became a pressing issue.

To achieve that, we are first learning the mechanisms by which antibiotics promote animal growth and other unintended impacts (Van Boeckel et al. 2015; Yang et al. 2017). Several hypotheses have been proposed: antibiotics could improve animal performance by reducing intestinal infection and bacterial toxin production; by preserving nutrients from microbial destruction; or by enhancing nutrient absorption via its mucus thinning effect*.* These antibiotic actions all link to their ability to influence microflora (Plata et al. 2022; Yang et al. 2017). Indeed, the role of the gut microbiome under antibiotics perturbation or in response to newly developed alternatives in pigs has been intensively studied (Kim and Isaacson 2015; Levast et al. 2014; Wang et al. 2019d). Whereas the effects of antibiotics on the host immunophysiology directly or via the collateral damage of the drug on commensal bacteria were less explored. From the perspective of general physiology, immunity is considered the gatekeeper for maintaining the molecular homeostasis of the whole body, especially to counteract pathogenic invasions. It is reported that chronic low-dose penicillin administration induces a global down-regulation of intestinal immunity in mice, such as reduced expression of transcription factors and cytokines important for Th17 cell function and genes related to defense responses (Cox et al. 2014). Early-life antibiotic exposures (ampicillin and neomycin) impair antibody responses to several vaccines in mice, including the failure of the Bacillus Calmette-Guerin vaccine (Lynn et al. 2018). Alternatively, antibiotics may affect host cell metabolism and their inflammatory signaling, thus resulting in changes in gut microflora (Zarrinpar et al. 2018). However, the exact nature of these interactions is still elusive.

Therefore, in search of alternatives to antibiotics in pig production, we focus on results and candidates that exhibit the potential to maintain or restore the physiological state of pigs and support their optimal growth and immune responses. In this review, we will briefly describe pig mucosal immunity as a physiological system in its functions, followed by discussions of the gut microbiota dynamics in swine during the weaning period. Furthermore, evidence of in-feed antibiotic alternatives with immuno-modulatory properties will be summarized in a non-exhaustive way highlighting probiotics, prebiotics, and phytobiotics. Finally, we will conclude the review with our immunophysiological perspective on the status of the field and ask questions important for its future perspective.

## The mucosal immunity of pigs

The intestinal mucosal immune system contains multiple layers, each composed of phenotypic diverse and functional plastic cell subsets. The inter-dependent cellular network must act in concert while each component endows with specialized and complementary functions (Ansaldo et al. 2021; Collins and Belkaid 2022). The mucosal sites of the gastrointestinal (GI) tract are constantly exposed to high loads of antigens, including the microbiome, dietary components, and other environmental factors. As a result, the host responses to oral delivery of antigens should be tightly regulated by the local intestinal mucosal immune system to prevent inflammation and other damages (Lavelle and Ward 2022). There are more than 80% similarities in analyzed parameters of the immune system between pigs and humans (Pabst 2020). Indeed, pigs are invaluable animal species not only for their economic significance but also for the high resemblance (Käser 2021; Pabst 2020). It has led to an increasing interest in using the pig as a biomedical model, exploring their digestive immunophysiology and nutritional regulation in health and diseases (Wylensek et al. 2020). However, the porcine intestinal immune cell landscape is less defined. Enhanced characterization of the porcine mucosal immunity, especially immune cell functionality, may provide insights into the mechanisms and outcomes of feed antigens added as alternatives to antibiotics in pig production (Peng et al. 2021; Wiarda et al. 2022).

### The organization of gut-associated lymphoid tissues

**Fig. 1:**
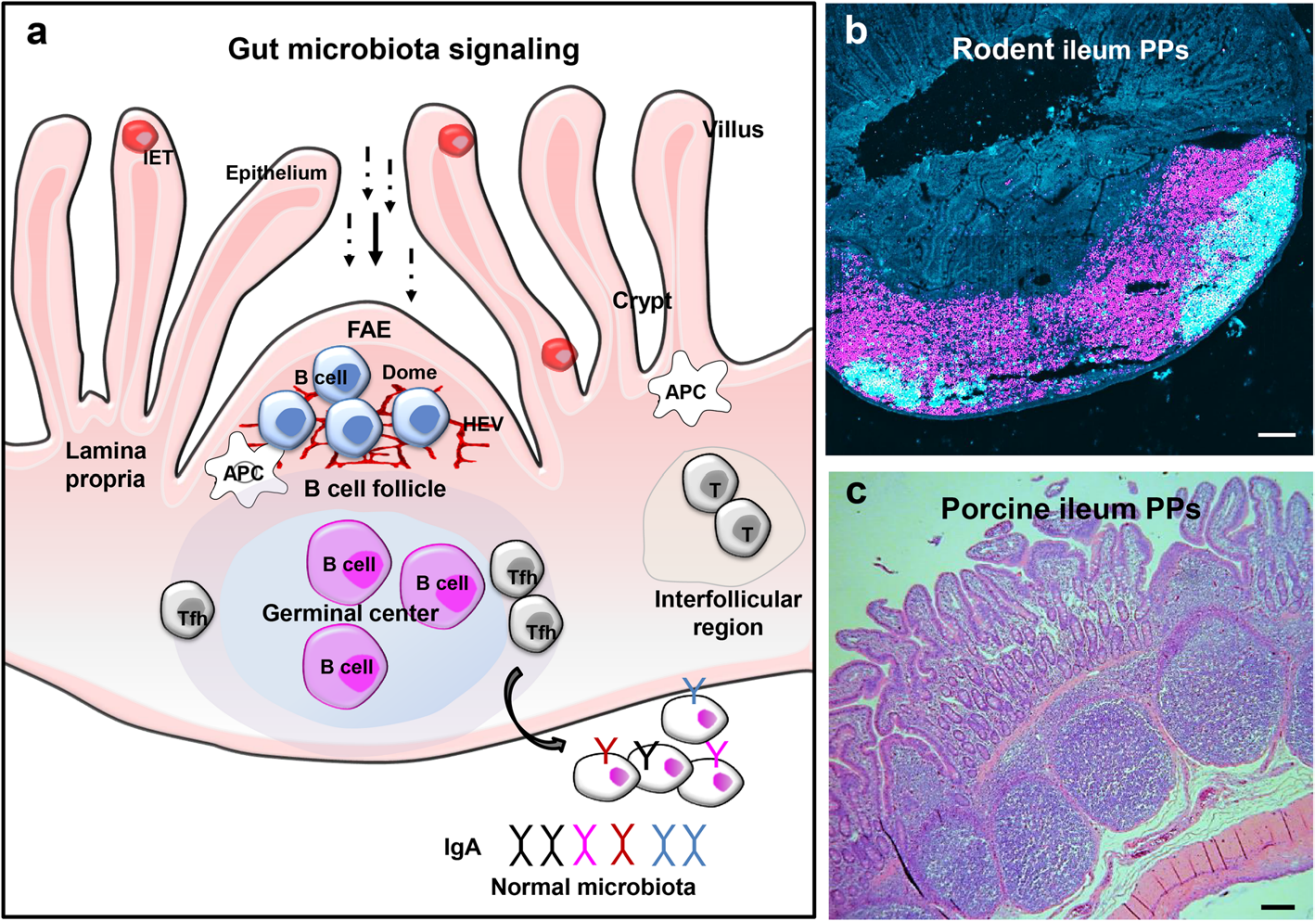
The organization of Peyer’s patches (PPs). (**a**) Schematic representation of PPs and the immune cell distribution. (**b**) Immunohistochemical staining of rodent ileum PPs with anti-GL7 (cyan) for germinal center and anti-B220 (magenta). (**c**) Histological image of porcine ileum PPs with H&E staining. Scale bars = 100 μm. FAE, follicular associated epithelium; IEL, intraepithelial lymphocyte; HEV, high endothelial venules

The gut-associated lymphoid tissues (GALT) in pigs comprises two types of Peyer’s patches (PPs), a number of isolated lymphoid follicle (ILF) and the intestine-draining lymph nodes (Fig. 1). It is the primary inductive site of antigen recognition, elimination, and antigen-specific B and T cell reactions (Furukawa et al. 2020). Unlike the evenly distributed PPs throughout the small intestinal wall of mice, the porcine PPs in the jejunum and upper ileum are discrete, while the terminal ileal PPs present as a continuous strip (Haley 2017). Both types of PPs are covered by follicle-associated epithelium (FAE), including the specialized M cells, to take up intestinal luminal antigens and transfer them to MHCII expressing antigen-presenting cells (APCs) beneath. In the follicular and interfollicular regions of PPs in pigs are abundant CD20^+^ B cells and a few T cells, with significantly higher numbers in the ileum than in the jejunal compartments (Nochi et al. 2020). Based on the expression of antibody isotype, the porcine PPs CD20^+^ B cells are further divided into CD20^+^IgM^-^ cells in the marginal zone and the CD20^+^IgM^+^ phenotype in the central zone (Furukawa et al. 2020). Unlike most follicles deep in the paracortex, the porcine GALT presents an inverted structure. The tissue is composed of cortical areas and paracortex, with internally placed germinal centers and a medulla located on the outside of the node. In these structures, lymphocytes exit via high endothelial venules directly into the blood instead of via efferent lymph vessels (Mair et al. 2014). However, the functional relevance of these inverted lymph node structures remains elusive.

In conventionally-raised piglets, the number of PPs follicles may peak at one or two months of age, reaching 75,000. The number then declines while these PPs gain structure maturity at six months. Microbial exposure is not required for porcine jejunal or ileal PPs organogenesis. Similarly, in humans, GALT is already in development at the embryonic stage, which could prepare the neonates to establish robust protective immunity at birth (Furukawa et al. 2020). Although not necessary for PPs development, commensal microbiota is vital for the immunological maturity of porcine GALT in young adults, priming appropriate T and B cell activation, and IgA responses (Furukawa et al. 2020). In germ-free or gnotobiotic pigs, B cells in PPs preferentially differentiate into IgM^+^ than IgA^+^ cells. Their GALT shows poor post-natal development with limited IgA repertoire diversification (Butler and Wertz 2012).

### The immunophysiological roles of IgA induction and production

The primary task of GALT is IgA induction, consequently controlling intestinal mucosal secretory IgA (SIgA) responses (Liu et al. 2021; Rollenske et al. 2021). SIgA is the dominant class of antibodies secreted into mucosal barrier sites at gram levels. With the introduction of gut microbiota diversity, we are also learning that SIgA plays a broad range of immunophysiological roles in host animals (Huus et al. 2021). Firstly, SIgA acts through agglutination and immune exclusion to limit bacterial colonization and prevent pathogen invasion. One example is SIgA binding to *Salmonella enterica* to inhibit its type III secretion and spread systemically (Richards et al. 2021). Furthermore, Bansept and coworkers have demonstrated that IgA cross-links bacteria into clusters as they divide, where fast-replicating ones are potential pathogens thereby induce more immune reactions and vice versa (Bansept et al. 2019). Secondly, SIgA can directly affect bacterial gene expression and epitope production, including dampening the expression of their motility genes. In contrast, SIgA may also promote bacterial colonization by forming adhesive sites/biofilm and influencing metabolic interactions between bacteria. For instance, IgA responses can be co-opted by commensal Bacteroidetes to facilitate bacterial adherence via regulating their gene expression of polysaccharides utilization (Donaldson et al. 2018). By estimation, a single bacterium in the intestinal lumen is coated by up to 800 SIgA molecules (Bansept et al. 2019). Based on the parallel generic and unique epitope-specific effects of SIgA, it can target a single microbe with distinct effects on microbial carbon-source uptake, bacteriophage susceptibility, motility and membrane integrity, thus differentiating the commensals from the pathobionts (Rollenske et al. 2021).

In swine, the placenta does not allow the transportation of maternal IgA and other proteins during fetal development. Thus, the acquisition of passive immunity (systemic and mucosal) in pigs occurs only after birth from colostrum and through lacteal uptake until weaning. It is a critical time to obtain ‘natural antibodies’ to survive and to establish tolerance to dietary proteins and commensal microbes (Butler and Wertz 2012; Virdi et al. 2019). In germ-free conditions, piglets are immuno-unresponsive, where neither T cell-dependent nor independent IgA responses can be formed appropriately. However, the provision of live bacteria or purified microbe-associated molecular pattern (MAMP) is sufficient to stimulate both types of T-cell reactions and the development of adaptive immunity in these animals, which is reviewed in detail elsewhere (Butler and Wertz 2012). In this regard, the dietary addition of probiotics, organic acids, bioactive plant components, and spray-dried plasma as adjuvants to increase intestinal IgA secretion has been widely tested in pig production (Krimpen et al. 2014). These bring about a now-popular idea that SIgA-microbiota interaction represents a significant axis of host homeostasis. And oral feed-based immune regulation can be a promising strategy in post-weaning piglets’ management (Virdi et al. 2019).

### The intestinal barrier

Although produced by the adaptive immune system, SIgA also constitutes part of the ‘natural defense’ of the gut barrier, cross-linking bacteria as they divide, thus controlling their cluster and interaction with epithelium (Saalmüller and Gerner 2016). The intestinal mucosa is a highly stratified system containing multiple layers. And maintaining the integrity of this system is of utmost importance in health and disease (Suzuki 2020). The first line of defense against extraordinary microbial pressure is the bilayer of mucus. This immune defense line of the intestine is formed by the continuous secretion of mucin by goblet cells. In the rodent colon, the mucous gel is divided into two layers: the loosely adherent mucus layer enchained with SIgA, which serves as a unique niche for some bacterial groups such as *Akkermansia muciniphila* and the abundant *Bacteroides* species; while the firmly adherent mucus layer is responsible for keeping commensal microbiota from the epithelium monolayer (Liu et al. 2019). During inflammation, malnutrition, or antibiotic perturbation, the mucus bilayer may be disrupted and thin out, allowing bacteria to invade epithelial cells and impair the barrier function (Paone and Cani 2020). However, whether the porcine mucus layer is organized in the same manner and plays similar roles in pig health is unclear. Finally, the epithelial layer serves as the last line of defense and is maintained by tight-cell junctions with a surprisingly complex protein composition. This paracellular diffusion barrier is formed predominantly by three transmembrane proteins, occludin, claudins, and junction adhesion molecule proteins (Suzuki 2020). These are associated with an array of peripheral membrane proteins, such as ZO-1 and heat shock proteins (HSPs), which join together to stabilize the cytoskeletons of adjacent cells and control paracellular permeability (Liu et al. 2014a). The latter chaperone proteins of epithelium are also emerging immuno-regulatory molecules, closely linked with gut microbiota alterations (Liu et al. 2014a; Liu et al. 2022a).

Lastly, interactions between the epithelial layer and luminal bacteria can provide the signals directing the type of immune responses in the lamina propria by altering the cytokine microenvironment (Peng et al. 2021). At weaning, the intestinal bacterial community composition of piglets fluctuates dramatically and induces a transient increase of epithelium permeability. This would facilitate the passage of toxic substances and pathogens at the mucosal barrier, and stimulate a vigorous immune response (Moeser et al. 2017; Pluske et al. 2018). In contrast, inhibition of this weaning reaction by eliminating all bacterial activity with antibiotics would lead to pathological imprinting of immunity (Al Nabhani et al. 2019).

### Other intestinal immune components

In addition to GALT, the intraepithelial T cells (IETs) and lamina propria lymphocytes are also important components of intestinal mucosal immunity. As the earliest immune cells populating the intestine, IETs can provide immune protection during early life by releasing cytotoxic molecules and/or antimicrobial peptides (Wiarda et al. 2020). The porcine IETs belong to both γδ and αβ T cell lineages preferably with CD8^+^ phenotype (*i.e.*, CD3^+^CD2^+^CD8α^+^ γδ T and CD3^+^CD4^−^CD8α^+^ αβ T cells) and a few CD3^+^CD2^+^CD8α^−^ γδ T cells. The number of IETs increases over time along with the gut microbiota colonization and becomes comparable to adult pigs at two months of age (Wiarda et al. 2020). While their distribution is site-dependent, with more in the ileum (30 per 100 enterocytes) than in the jejunal compartment (20 per 100 enterocytes) (Wiarda et al. 2020; Wiarda et al. 2022). In germ-free piglets, the IET numbers are low, indicating a lack of microbial antigens stimulation and a compromise of immunity (Potockova et al. 2015).

In contrast to IETs, the porcine lamina propria T cells are predominantly CD4^+^ phenotype and play a central role in animal health and against infectious diseases, which is thoroughly discussed elsewhere (Käser 2021). In pigs, the lamina propria effector T cells are preferably recruited to their site of origin during recirculation, by molecular machinery of integrin α4β7, addressin MAdCAM, chemokines CCL25 and CCL28, *etc.* (Peng et al. 2021). Furthermore, this effector site also serves for the regulation of IgA responses, containing the majority of IgA-producing plasma cells of pigs. In lamina propria, plasma cells and plasma cell precursors are more often present in the crypts. This pool of IgA-producing plasma cells in the pig intestine is clearly associated with responses to commensal microbiota (Nochi et al. 2020; Wiarda et al. 2022).

## The porcine gut microbiota and The Hygiene Hypothesis

### The gut microbiota in swine and its association with antibiotics

The mammalian gut microbiota is a very complex ecosystem with high diversity, evolving with time and changes according to the composition of diet. It influences many aspects of intestinal physiology, including fermenting dietary fiber to produce short-chain fatty acids (SCFAs), regulating lipid metabolism and generating secondary bile acids, forming a microbial barrier to exclude pathogenic bacteria, and modulating the immune system to protect pigs against infections (Gresse et al. 2017; Wang et al. 2019d). With the advent of 16S rRNA amplicon sequencing and metagenome-assembled genomes analysis, we are uncovering diversity within pig-specific bacterial groups, including *Lactobacillus* spp., *Streptococcus* spp., *Clostridium* spp., *Fusobacterium* spp. and even new genera that yet to be defined (Wylensek et al. 2020; Yang et al. 2022). In pig production, weaning is one of the most critical events which frequently leads to intestinal disorders and antibiotic therapies, raising concerns for the economy and public health (Massacci et al. 2020). It is reported that the predominant bacterial groups of piglets after birth are *Lactobacillus* spp., followed by *Escherichia/Shigella*. Soon enough, the microbial community of suckling piglets is represented by *Lactobacillus*, *Escherichia/Shigella*, as well as *Fusobacterium*, *Bacteroides*, and *Megasphara* (Chen et al. 2017). At weaning, piglets face sudden milk withdrawal, changes in social conditions and physical environments, and ingest solid feed for the first time in life (Gresse et al. 2017). During the weaning transition, the relative abundance of *Lactobacillus* spp. decreases, whereas anaerobes such as *Clostridium* spp. and *Prevotella* spp. become more abundant. The number of *Lactobacillus* spp. continues to decline during the post-weaning time, among which the number of *L. amylovorus* and *L. reuteri* decrease the most in the porcine ileum (Call et al. 2018), while *Clostridium* spp. and *E. coli* eventually colonize the intestine of piglets (Alain et al. 2014; Gresse et al. 2017). At this time, not only the intestinal microbiota is unstable, but also the animals still have an immature immune system and low digestive capacities, together profoundly impact pig growth performance and their susceptibility to infectious diseases (Luise et al. 2021b; Luo et al. 2022). Meanwhile, introducing fiber and protein to post-weaning diets is challenging for the piglet’s intestine, which may induce gut microbiota dysbiosis and inflammation. For instance, high protein inclusion of up to 20% can increase bacterial protein fermentation and the production of potentially toxic metabolites such as ammonia and amines in the large intestine, thus increasing the risk of diarrhea in weaning piglets (Luise et al. 2021a). Therefore, in the animal husbandry, oral administration of antibiotics, especially during weaning periods becomes the most common practice worldwide (Van Boeckel et al. 2015).

Although the mechanisms by which AGPs promote animal growth are not fully understood, it is at least partly mediated by changes in gut microbiota, as the effects diminished in germ-free animals (Plata et al. 2022; Yang et al. 2017). Direct effects of antibiotics on the intestinal bacteria include inhibiting opportunistic pathogens and reducing competition for nutrients (Rahman et al. 2022). However, antibiotic administration also has negative impacts on the commensal bacterial populations. Gao et al. (2018) revealed that ceftriaxone sodium administration causes tens of multiple-folds decrease of *Lactobacillus* and *Bifidobacterium* spp. in the porcine ileum (Gao et al. 2018). The relative abundance of lactic acid bacteria was also found to decrease by amoxicillin treatment in the jejunum of weaned piglets (Bosi et al. 2011). Furthermore, ASP250 (chlortetracycline, sulfamethazine, and penicillin) supplementation in feed results in reductions of intestinal *Coprococcus*, *Succinivibrio*, *Streptococcus*, *Treponema*, and *Turicibacter* spp. in pigs (Allen et al. 2011). In these studies, a simultaneous increase in *Escherichia* population is observed regardless of antibiotic classes used. In addition to causing such gut microbiota dysbiosis, the overuse of antibiotics eventually leads to resistance virulence factors to be found in gene families unique to the swine fecal metagenome, including sequence homology to genes in the dominant bacterial populations (*e.g.*, Bacteroidetes and *Clostridia*) (Lamendella et al. 2011), and dysregulation of the immune system (Bosi et al. 2011; Schokker et al. 2014).

### The Hygiene Hypothesis in livestock

Indeed, in mammals, the gut commensal microbiota dictates the host's immunophysiology. For instance, bacteria can stimulat+e B cell division in GALT via TGFβ signaling (Liu et al. 2021) or promote intestinal epithelium differentiation and integrity via a proliferation-inducing ligand (APRIL) regulation (Allaire et al. 2018). Without appropriate signals from the microbiota, such as inhibition of weaning reaction by antibiotics, skewed immune responses can take place (Al Nabhani et al. 2019). It is what “The Hygiene Hypothesis” entails, describing how host immunity and their microbiota interact in a continuously modernizing environment. In the livestock production system, the evolution of porcine gut microecology is consistent with the basic idea of “The Hygiene Hypothesis” in humans: improved hygiene conditions, reduced exposure to environmental microorganisms in early life, and frequent use of antimicrobial drugs drive immune dysregulation (Pfefferle et al. 2021). In addition to antibiotic overuse in swine, the dietary structure/formulation and feeding management have also changed significantly during modernization, resulting in a decline of bacterial community diversity, a complete loss of some bacterial taxa and their function, and a persistent impact on animal immunophysiology (Gao et al. 2019; Gresse et al. 2017; Yang et al. 2022). For instance, long-term use of antibiotics could alter the intestinal expression of toll-like receptors (TLRs) and PPs cellularity (Grasa et al. 2015), subsequently one’s tolerance to the commensal microbiota (Kim et al. 2017). It may also affect animal liver regeneration (Wu et al. 2015) and bile acid synthesis (Chen et al. 2022) and disrupt body composition, including fat depot (Li et al. 2021b). However, disparate findings are reported as antibiotics have a broad spectrum of activities.

## Alternatives to antibiotics in pig production: modulating the immunophysiology

Basic studies focusing on how antibiotic substitutes affect the physiology and immunology of livestock are scarce. As a monogastric, omnivorous large animal species, pig is ideal for studying the immuno-regulatory mechanisms of probiotics, prebiotics, and other natural feed/food additives and their applications (Dowarah et al. 2017). In the following, specific types of substrates that display immuno-modulatory properties in the health management of pig production are discussed.

### Probiotics

**Fig. 2:**
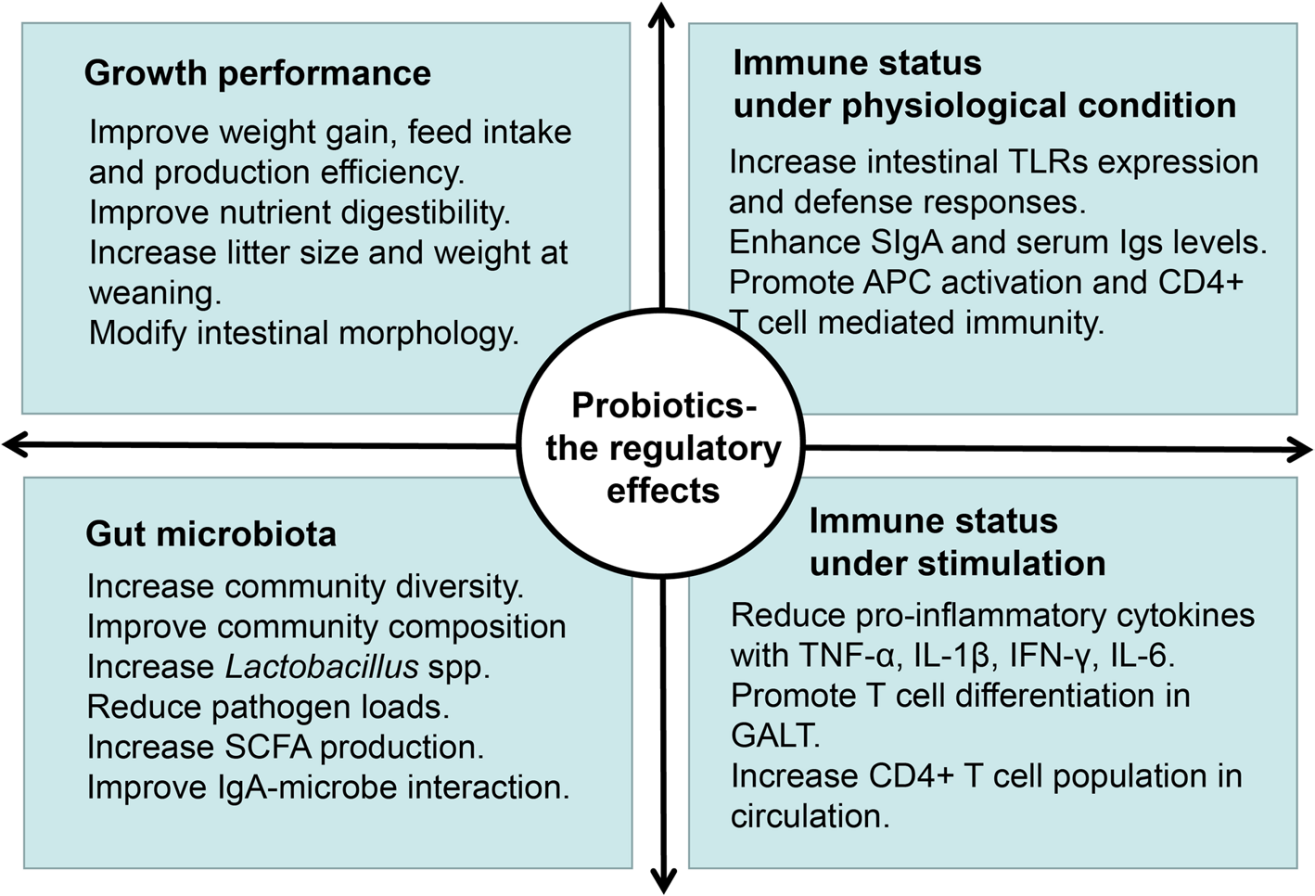
Schematic illustration of the regulatory effects of probiotics in swine

Probiotics refer to live microorganisms that can confer health benefits on the host when given in adequate amounts, such as restoring the gut microbiota homeostasis and improving the immunophysiological health of mammals (Salminen et al. 2021) (Fig. 2). They are superior to antibiotics as being safe for long-term administration and do not cause severe side effects (Virdi et al. 2019). Among the tested probiotics including lactic acid bacteria, *Bacillus* (*e.g.*, *B. subtilis*), *Enterococcus* (*e.g.*, *E. faecium*), *Streptococcus* (*e.g.*, *S. infantarius*), *Pediococcus* (*e.g.*, *P. acidilactici*), some butyrate-producing bacteria, yeast, Aspergillus, and Trichoderma, *etc.*, *Lactobacillus* species seem to have the highest potential to replace antibiotics in swine (Patel et al. 2015). Nevertheless, the question remains as to how the addition of a small population of ingested, transient bacteria could stir the gut microbiome and the host immune system. Our previous study in mice demonstrates that distinct B cell subsets in the PPs act as relays that sense, enhance, and transmit the *Lactobacillus reuteri* signals, thereby promoting IgA responses, which shapes the microbial community and protects against inflammation (Liu et al. 2021).

Indeed, one of the key effects of *Lactobacillus*-based probiotics in pigs is the modification of humoral immune responses through promotion of IgA production and suppression of pro-inflammatory cytokines (Table 1). In healthy piglets at the early stage of post-weaning, dietary supplementation of *Lactobacillus* spp. (*e.g.*, *L. plantarum*, *L. fermentum*, *L. delbrueckii* and/or *L. rhamnosus*) increased their serum IgA, IgM (Peng et al. 2022; Wang et al. 2019d; Wang et al. 2018), and IgG (Ahmed et al. 2014; Shin et al. 2019) concentrations, as well as enhanced the small intestinal SIgA production (Li et al. 2019; Yi et al. 2018). Whereas under pathophysiological conditions such as diarrhea or weaning stress, the circulation levels of TNF-α (Wang et al. 2019b), IFN-γ and IL-6 (Qiao et al. 2015; Yang et al. 2020) are substantially reduced by *Lactobacillus-*based probiotics in piglets. Consistently, the expressions of the former two pro-inflammatory cytokines are also decreased in the small intestinal mucosa of challenged piglets by *L. delbrueckii* (Chen et al. 2020) and by *L. plantarum* treatment (Tang et al. 2021), respectively.

**Table 1:** The immuno-modulatory activities of *Lactobacillus*-based probiotics in the health management of pig production

Probiotics	Dosage	Phase	Duration	Challenge	Key immuno-modulatory activities	References
*L. delbrueckii* CCTCCM 207040	5×10^9^ CFU/mL/pig	Suckling	21, 28 or 49 days	None	Increases IgA levels associated with promotion of jejunal and ileal IL-4, IL-10 production; promotes DC maturation and activation associated with TLR2/4-mediated NF-κB and MAPK signaling pathways; induces long-term effects of increasing serum IL-12, jejunal and ileal TNF-α levels	(Peng et al. 2022)
*L. plantarum* PFM 105	2×10^7^ CFU/g	Weaning	21 days	None	Increases serum IgM and the intestinal SIgA levels; increases serum IL-2, IL-10, and TGF-β levels	(Wang et al. 2019c)
*L. plantarum* ACCC 11016	1.6×10^9^ CFU/kg and FOS (1.5 g/kg)	Post-weaning	28 days	None	Increases plasma IgA and IgG concentrations	(Wang et al. 2018)
*L. reuteri avibro* or *B. subtilis* and *B. licheniformis*	10^10^ CFU/kg *L. reuteri avibro*; 3.2×10^9^ CFU/kg *Bacillus*	Post-weaning	28 days	*Salmonella enterica serovar Typhimurium*, *E. coli*	Increases the serum IgG levels	(Ahmed et al. 2014)
*L. plantarum* JDFM LP11	2.5×10^7^ CFU/mL	Post-weaning	28 days	None	Down-regulates immune associated genes *BPI*, *RSAD2*, *SLPI*, *LUM*, *OLFM4*, *DMBT1* and *C6*, promotes serum IgG levels	(Shin et al. 2019)
LAB CCTCC M 207040	5×10^9^ CFU/mL	Suckling	21, 28 or 49 days	None	Increases jejunal and ileal SIgA concentrations, small intestinal IL-12, CX3CL1 and MIP3α levels; increases the small intestinal COX2 and iNOS expression	(Li et al. 2019)
*L. reuteri* LR1	5×10^10^ CFU/kg	Post-weaning	14 days	None	Increases ileal SIgA, IL-22 and TGF-β levels; increases TLR2, TLR4, β-defensin 2 and protegrin 1-5 gene expression	(Yi et al. 2018)
*L. fermentum* and *P. acidilactici*	1.6×10^9^ CFU/g	Post-weaning	28 days	None	Decreases serum IL-6, IFN-γ and IL-1β levels	(Wang et al. 2019c)
*L. acidophilus*	5 x10^10^ CFU/g	Post-weaning	28 days	*E. coli* LPS	Increases serum IL-10 concentrations and reduces serum TNF-α and IFN-γ levels	(Qiao et al. 2015)
*L. johnsonii* L531	1.0×10^10^ CFU/day	Post-weaning	18 days	*S. Infantis*	Reduces jejunal lipocalin 2 production; increases the number of CD4^+^CCR6^+^ T cells in MLNs; reduces ileal villus loss and villus length shortening	(Yang et al. 2020)
LAB CCTCC M 207040	2.0 × 10^10^ CFU/g	Post weaning	28 days	LPS	Decreases intestinal TNF-α and IL-1β concentrations, and increases intestinal IL-10 levels	(Chen et al. 2020)
*L. plantarum* CGMCC 1258 or *L. reuteri* LR1	5×10^10^ CFU/kg	Post-weaning	42 days	None	Reduces the small intestinal expression of IL-1β, TNF-α and IFN-γ; increases the TGF-β expression, and promotes TLRs-related pathways to regulate the SIgA secretion	(Tang et al. 2021)
*L. plantarum* ZLP001	10^9^ CFU/g	Post-weaning	28 days	None	Increases the jejunal and ileal host defense peptide expressions via TLR2, ERK1/2/JNK and c-jun/c-fos signaling pathways	(Wang et al. 2019a)
*L. buchneri* NLRI-1201, *L. plantarum* NLRI-101, *L. casei* DK128 with FOS	1.2×10^8^ CFU/g, 1.6×10^8^ CFU/g, 1.4×10^8^ CFU/g	Growing	3 months	None	Upregulates the intestinal TLR signaling, NOD1and NOD2 expression; reduces the mRNA and protein expression levels of IL-1 β, TNF- α, IL-6, and IFN- γ in the colon	(Kim et al. 2021)
*L. rhamnosus* GG ATCC 53103	6×10^9^ CFU/mL/day	Post-weaning	30 or 45 days	None	Increases CD3^+^CD4^+^ T lymphocytes in MLN, jejunal, ileal PPs and LP; increases ileal villus length	(Shonyela et al. 2020)
*L rhamnosus* GG ATCC 53103 and *B lactis* Bb12	10^5^ CFU/pig/time	Suckling	28 or 35 days	HRV Rotaviruses	Increases intestinal CD25^+^Foxp3^+^ Tregs, CD3^+^CD8^+^IFN-γ^+^ T cells numbers and serum TGF-β levels; reduces serum IL-8, IL-17, biliary TNF-α and IL-12 levels	(Chattha et al. 2013)
*L. reuteri* I5007	1×10^8^ CFU/mL	Suckling	24 days	None	Increases the expression of jejunal pBD2, colonic pBD2, pBD3, pBD114 and pBD129 associated with the increase of butyrate concentration and PPAR-γ and GPR41	(Liu et al. 2017)

*CFU* colony-forming unit, *DC* dendritic cell, *IL* interleukins, *TLR* toll-like receptor, *FOS* fructooligosaccharides, *LPS* Lipopolysaccharides, *MLNs* mesenteric lymph nodes, *PPs* Peyer’s patches, *LP* lamina propria

Another key effect of *Lactobacillus*-based probiotics on pigs is the modification of intestinal cellular immune responses specific or non-specific to bacterial antigens (Maldonado Galdeano et al. 2010; Patel et al. 2015). Although it is not fully understood how immune activation or tolerance is achieved in response to the probiotic bacteria, interactions between MAMPs and pattern recognition receptors (PRRs) of the host intestinal cells are involved (Liao and Nyachoti 2017). One such mechanism by which *Lactobacillus*-based probiotics suppress inflammation has been suggested to be initiated by TLR2 (Peng et al. 2022; Wang et al. 2019a), TLR4 (Peng et al. 2022; Tang et al. 2021; Yi et al. 2018), or NOD1 activation (Kim et al. 2021) through NF-κB or MAPK signaling pathways in the jejunum and ileum of pigs. Thereafter, the recognition of probiotic antigens leads to regulations of cell-mediated immune responses in the porcine intestine. A recent study has demonstrated that oral gavage of *L. delbrueckii* promotes the intestinal professional APCs’ maturation of suckling piglets. The corresponding chemokine CCL20-chemokine receptor CCR6 signaling was upregulated upon probiotic stimulation, thereby mediating a strong APCs activation in the porcine small intestine. Intriguingly, this modulation of local intestinal immune responses by *L. delbrueckii* produced a long-lasting effect in piglets until after weaning (Peng et al. 2022). Subsequently, a better primed T cell reaction may be expected in response to *Lactobacillus* probiotic treatments. In *Salmonella infantis*-induced diarrhea in piglets, *L. johnsonii* administration results in the expansion of CCR6^+^CD4^+^ T cells in GALT while inhibiting systemic inflammation (Yang et al. 2020). Furthermore, Shonyela and co-workers (2020) reported that orally administered LGG can enhance T cell differentiation with an increased CD4^+^ T cell population in the porcine GALT (Shonyela et al. 2020). In a neonatal piglet model of human rotavirus infection (HRV), LGG-based probiotic combination attenuates disease by promoting both innate MHCII-expressing APC and Th1 immunity via TLR expression (Chattha et al. 2013). It is also shown that *L. reuteri* strongly upregulates the expression of β-defensin 2 and protegrin 1-5 in the small intestinal mucosa of healthy piglets when compared to their antibiotic-treated counterparts (Yi et al. 2018). In contrast, transcriptome analysis reveals that *L. plantarum* down-regulates ileal gene signatures associated with innate defense responses and promotes gut development in weaned pigs (Shin et al. 2019). Interestingly, a study in neonatal piglets suggests that the *Lactobacillus-*enhanced natural defense response is associated with intestinal immuno-metabolism alterations (Tang et al. 2021), such as peroxisome proliferator-activated receptor-γ (PPAR-γ) activation and SCFAs production (Liu et al. 2017). It is shown in drosophila that *L. plantarum* modulates the host capacity of food-derived protein digestion by enhancing their intestinal proteolytic enzyme activity via NF-kB signaling, whereas the pathway can be highjacked by pathogen infection (Erkosar et al. 2015; Park et al. 2015). Nevertheless, it remains elusive how the hosts prioritize immuno-metabolism in response to commensal microbes versus pathogenic bacteria.

Although antibiotics and *Lactobacillus* are both effective growth promoters and prophylaxis to prevent infection in livestock, unlike probiotics, in-feed antibiotics cannot be used as immune enhancers in healthy pigs, if not the opposite. It is why we address *Lactobacillus* spp. and other antibiotic alternatives by looking through the lens of immunophysiology in the current review, as it may open up new avenues for health management in pig production. However, there are several weak points of probiotics: firstly, a lack of consistent effects on porcine immunity and understudied regulatory mechanisms (Mingmongkolchai and Panbangred 2018); secondly, lacking viability and efficacy tests and established application protocol in farm conditions (Barba-Vidal et al. 2019). It is partly due to the extremely high complexity and diversity of gut microbiota in pigs, like humans (Yang et al. 2022), thus the varied responses to one probiotic. The newly developed pipeline of probiotics investigations focuses on thorough characterizations of the porcine commensal bacterial community first, then precisely identifying the potential probiotic candidates and strategically applying them into different scenarios (Hu et al. 2018; Wang et al. 2022). Towards this end emerges fecal microbiota transplantation (FMT) as an alternative strategy, introducing the microbiome to improve gut health in farm animals (Hu et al. 2018; Rouanet et al. 2020). The approach has been actively used in poultry and is arising in the health management of swine (Canibe et al. 2019).

### Prebiotics

Prebiotics are substrates that can promote the growth of specific groups of commensal bacteria and/or their metabolism, and confer health benefits in the host (Salminen et al. 2021). Taking the classic prebiotic inulin for example, it is proven to increase the population of probiotic *Lactobacillus* spp. and *Bifidobacterium* spp., and promote lactic acids and SCFA production with a reduced pH in the intestine, thus suppressing pathogenic bacteria growth (van der Aar et al. 2017). In addition to SCFA promotion, the proposed mechanisms of prebiotic action also include blocking receptor sites for bacterial adhesion and immuno-modulation, *etc.* (Bai et al. 2022; Cunningham et al. 2021). In particular, prebiotics may improve the immune barrier by interacting with PRRs on intestinal epithelium and/or leukocytes in the GI tract, as well as with other immune components such as IgA (Table 2).

**Table 2:** The immuno-modulatory activities of prebiotics in the health management of pig production

Prebiotics	Dosage	Phase	Duration	Challenge	Key immuno-modulatory activities	References
Xylooligosaccharide	500 mg/kg	Post-weaning	28 days	None	Promotes cecal HSPs accumulation; suppresses IL-6, IL-8 expression via microbiota-derived metabolites activated G-protein coupled receptors or inhibited histone deacetylases; suppresses the expression of signal molecules-associated antigen cross presentation in the process of SIgA production	(Tang et al. 2022)
Galactomannan-oligosaccharides or Chitosan	0.2% (w/w) or 250 mg/kg	Post-weaning	14 days	None	Increases *IL-1β* expression in jejunum and lymph nodes; increases serum levels of IL-1β, IL-2, IL-6, IgA, IgG and IgM	(Yin et al. 2008)
Isomaltooligosaccharides	0.2%, 0.4%, 0.6% or 0.8% (w/w)	Post-weaning	28 days	None	Increases IgA, IgM, IgG and IL-2, decreases IL-6 in the serum	(Wang et al. 2016)
5% resistant potato starch	5% (w/w)	Post-weaning	21 days	None	Increases the abundance of Tregs in the cecum associated with increased luminal IgA concentration and expression of IL-6 and DEF1B	(Trachsel et al. 2019)
Lactulose, inulin, sugar beet pulp, and wheat starch combined	20 g/kg, 7.5 g/kg, 50 g/kg and 50 g/kg	Post-weaning	10 days	None	Upregulates the colonic IL-6 mRNA expression	(Pié et al. 2007)
Alginic acid oligosaccharide	100 mg/kg	Post-weaning	21 days	None	Increases serum IL-10, IgG and IgA concentrations; increases the small intestinal SIgA content	(Wan et al. 2016)
β-galactomannan or mannanoligosaccharide or monosaccharide D-Mannose	10 μg/mL	IPI-2I *in vitro*	30 min	*S. Typhimurium*	β-galactomannan or mannanoligosaccharide reduces IL6 and CXCL8	(Badia et al. 2013)
Citrus peel pectin	5% (w/w)	Post-weaning	28 days	None	Increases IL-22, decreases IL-17 levels in serum; increases the expression of IL-10 in the jejunal mucosa, and down-regulates IL-1β, IL-6, IL-8, IL-17 and TNF-α expression via tryptophan metabolite-activated AhR/IL-22/STAT3 signaling pathway	(Dang et al. 2023)
Lactulose	1% (w/w)	Post-weaning	17 days	*S. Typhimurium* 4/74 or *S. Enteritidis* P125109	Increases serum levels of IgM, IgG and IgA specific to *S. Typhimurium* and cross-reacting antibodies to *S. Enteritidis.*	(Naqid et al. 2015)
Isomaltooligosaccharides	6 g/kg	Post-weaning	28 days	None	Increases serum IgG levels	(Wu et al. 2017)
Chicory, mannan oligosaccharides, or chitosan	0.1%, 0.1% or 0.02% (w/w)	Post-weaning	28 days	None	Does not alter serum IgA concentrations	(Li et al. 2016)
Rapeseed meal fermented with *B. subtilis* 87Y	8% (w/w)	Growing	From 28 days of age to a body weight of about 35 kg	None	Increases lymphocyte counts and IgG, IgA and IgM levels in blood	(Czech et al. 2022)

HSP, heat shock proteins

The most commonly used prebiotics in swine are dietary fiber fractions and non-digestible oligosaccharides derived from plants. It includes arabinoxylans, pectin, xyloglucans, resistant starch (Azad et al. 2020; Bach Knudsen et al. 2017), fructo-oligosaccharides, lactulose, raffinose, maltodextrin, mannitol, galactooligosaccharide and inulin (Williams et al. 2019). Dietary supplementation of chicory-pectin to newly weaned piglets has been shown to improve their growth performance and enhance the cytoprotective HSPs expression in the epithelium, implying an improved gut function (Liu et al. 2014b). A recent study found that prebiotic xylooligosaccharide also induces HSPs accumulation in the intestinal mucosa of weaned piglets, while suppressing their IL-6 and IL-8 expression through G-protein coupled receptors (Tang et al. 2022). Accordingly, results of several studies have demonstrated that prebiotic supplementations reduce pro-inflammatory interleukin production and increase anti-inflammatory cytokine levels in pigs (Pié et al. 2007; Trachsel et al. 2019; Wan et al. 2016; Wang et al. 2016; Yin et al. 2008). Galactomannan-oligosaccharides and Chitosan additions upregulate IL-1β expression in the porcine jejunum and lymph nodes, and increase IL-1β, IL-2 and IL-6 in the serum (Yin et al. 2008). In contrast, β-galactomannan and mannanoligosaccharide supplements reduce IL-6 and CXCL8 levels against *Salmonella* infection *in vitro* (Badia et al. 2013). Furthermore, in pigs fed diets with citrus-pectin, the jejunal IL-10 expression was increased while the expression of IL-1β, IL-6, IL-8, IL-17 and TNF-α was decreased, likely through the gut microbiota-derived tryptophan-activated AhR/IL-22/STAT3 signaling pathway (Dang et al. 2023). There is also increased production of antibodies in the GI tract as well as in circulation of pigs fed diets with prebiotics. Lactulose can act as a prebiotic in weaned piglets challenged with *S. typhymuriun* by enhancing pathogen specific IgM, IgG and IgA responses (Naqid et al. 2015). In healthy pigs, prebiotic isomaltooligosaccharides (Wang et al. 2016; Wu et al. 2017) and fermented rapeseed meal increase IgA, IgM, and IgG levels in circulation (Czech et al. 2022). In contrast, results of a study using chicory, mannan oligosaccharides, or chitosan as antibiotic alternatives for weaning pigs showed no effects on animal growth or serum IgA levels (Li et al. 2016).

Although the mechanisms by which prebiotics affect antibody responses are not fully understood, it is suggested they may act in concert with regulatory T cell (Treg) regulation or by altering the gut microbiome (Liu et al. 2018; Trachsel et al. 2019). Indeed, the beneficial effects of a given prebiotic are proportional to its fermentability (Jiao et al. 2021), which is determined by gut microbiota maturity. On the other hand, the beneficial effects of a given prebiotic depends on its structural complexity (Beukema et al. 2020). *I.e.*, classic prebiotics such as inulin belongs to fructan with a degree of polymerization of 2 to 60. A wide range of bacteria in the distal part of the small intestine can utilize it due to its simple structure (Williams et al. 2019). It may explain the inconsistent prebiotic effects of inulin observed in managing gut health. In contrast, dietary fiber fraction with complex molecular structure can only be hydrolyzed by a few bacteria (Cantu-Jungles and Hamaker 2020). For instance, the unique pectin-*Bacteroides thetaiotaomicron* interaction makes the microbe highly targetable (Luis et al. 2018). Nevertheless, the use of prebiotics and their regulation are not well-established in swine.

### Phytobiotics

Phytobiotics, also known as phytochemicals or phytogenics, is an umbrella term for a diverse subset of plant-derived bioactive compounds. This comprises a list of more than 5,000 biologics identified from essential oil, fruits and vegetables, whole grains, herbs, and nuts, *etc.* (Li et al. 2021a). Although without nutritive values, numerous studies have shown growth-promoting effects of phytobiotic feed additives in swine (Bartos et al. 2016; Su et al. 2018; Wang et al. 2021b; Yang et al. 2019). Furthermore, Martel et al. (2020) have recently proposed that a fraction of phytobiotics that pass by the gut lumen may improve intestinal health and animal development by acting as prebiotics (Martel et al. 2020). The beneficial effects of phytobiotics on animals also include increase nutrient digestion, absorption, and secretion of intestinal mucus, saliva, and bile, anti-bacterial activities, anti-oxidation and immuno-modulation (Lillehoj et al. 2018; Valenzuela-Grijalva et al. 2017).

The search for phytobiotics as antibiotic alternatives began with herbal and spice extracts called essential oils (Helander et al. 1998). They are primarily a complex mixture of volatile, aromatic, and lipophilic organic compounds, including terpenes and phenylpropenes (Li et al. 2021a). Essential oils are well recognized for their broad-spectrum antimicrobial activities against pathogenic bacteria by damaging the cell walls (Omonijo et al. 2018). Acetone crude leaf extracts of *Syzygium legatii* and *Eugenia zeyheri* disrupt the cytoplasmic membrane of enterotoxigenic *E. coli* of swine origin and result in increased influx of propidium iodide (Famuyide et al. 2020). And thymol and cinnamaldehyde suppress *Clostridium perfringens* by altering the cell wall and lipids and proteins of the cell membranes, respectively (Gómez-García et al. 2020), reducing the risk of intestinal disorders. On the other hand, the very first proved botanical feed additive in livestock in Europe, an essential oil blend, is highlighted for its immunity enhancement effects (Lillehoj et al. 2018) (Table 3). Under physiological conditions, tea tree oil is shown to increase serum IgG levels and enzymes associated with anti-oxidant capacity (*e.g.*, superoxide dismutase and glutathione peroxidase) in pigs (Wang et al. 2021b). Likewise, the serum levels of IgG and IgM were also elevated in piglets fed diets with thymol and cinnamyl aldehyde supplementation (Su et al. 2018). While the same essential oil mixture reduces the plasma IL-6 and TNF-α concentrations and promotes lymphocyte proliferation (Li et al. 2012). Furthermore, dietary supplementation with *Macleaya cordata* (sanguinarine) reduces the blood concentrations of haptoglobin and serum amyloid A (Kantas et al. 2015). Local immuno-modulatory effects are also detected in young pigs administered essential oil. In addition to improve serum antibody reaction, tea tree oil is shown to increases liver IL-10 and decreases the IL-1β and TNF-α concentrations (Wang et al. 2021b). Hofmann et al. (2022) have demonstrated that oregano essential oil can increase expressions of *TJP1* (encoding tight junction protein ZO-1), Akt3, interferon signaling and CCL21 in the porcine small intestine (Hofmann et al. 2022). Capsicum oleoresin or turmeric oleoresin supplement up-regulates the gene signature of immune activation (*C1QA, C5, CCL25, CD46, CFB,* and *FCN2*), cell membrane integrity, and tight junctions in the porcine ileum mucosa; while garlic extract increases the expression of genes involved in fatty acid biosynthesis, defense response, and oxidation reduction in pigs (Liu et al. 2014c).

**Table 3:** The immuno-modulatory activities of phytobiotics in the health management of pig production

Phytobiotics	Dosage	Phase	Duration	Challenge	Key immuno-modulatory activities	References
Mediterranean herb oregano (*Origanum* *vulgare* L.)	2 g/kg, 4 g/kg or 8 g/kg	Post-weaning	35 days	LPS	Increases the proportion of lymphocytes in blood, tends to decrease CD4^+^ and CD8^+^ T cell subpopulation and their ratio	(Stelter et al. 2013)
18% thymol and cinnamaldehyde	0.01%	Post-weaning	35 days	None	Increases lymphocyte proliferation; reduces IL-6 and TNF-α in the plasma	(Li et al. 2012)
Tea tree oil	0.04%	Post-weaning	28 days	None	Increases serum IgG and liver IL-10 levels; decreases liver IL-1β and TNF-α concentrations	(Wang et al. 2021b)
13.5% thymol and 4.5% cinnamyl aldehyde	50 ppm, 100 ppm, or 200 ppm	Post-weaning	21 days	None	Increases serum IgG and IgM levels	(Su et al. 2018)
Commercial oregano essential oil	112.5 mg/kg	Post-weaning to fattening	From 28 days of age to 6 months	None	Upregulates jejunal *IFN-ε*, *IFN-ω* gene expression; upregulates ileal *CCL21* gene expression	(Hofmann et al. 2022)
Capsicum oleoresin, garlic botanical or turmeric oleoresin	10 mg/kg	Post-weaning	9 days	None	Capsicum oleoresin and turmeric oleoresin increase the gene expressions related to immune activation (*e.g.*, C1qa, C5, CCL25, CCR9 and TLR-2); garlic botanical up-regulates the gene expression related to defense responses (*e.g.*, LYZ, IRG6 and C9)	(Liu et al. 2014c)
Fumaric, citric, malic and sorbic acids) plus with thymol, vanillin and eugenol	1 g/kg or 2 g/kg	Post-weaning	21 days	ETEC F4 (K88^+^)	Reduces serum TNF-α, IL-6 and IL-10 concentrations	(Xu et al. 2020b)
Cinnamaldehyde, thymol, citric acid, sorbic acid, malic acid and fumaric acid	1 kg/ton	Post-weaning	28 days	*E. coli* and *S. aureus*	Increases serum complement 4 concentrations; increases serum IgG and IgM levels on d 14	(Yang et al. 2019)
Panax ginseng, dioscoreaceae opposite, atractylodes macrocephala, glycyrrhiza uralensis, ziziphus jujube and platycodon grandiflorum	0.1% or 0.3%	Post-weaning	28 days	None	Increases the respiratory burst and Salmonella-killing ability of polymorphonuclear leucocytes	(Huang et al. 2012)
*Origanum vulgare* L.	0.2%	Growing	190 days	Outdoor rearing	Ameliorates pro-inflammatory pathways by down-regulating HSP90, NFKB1 and STAT3 expression	(Cappelli et al. 2021)
Cranberry extract, encapsulated carvacrol, yeast-derived products and extra vitamins A, D, E, and B complex	1 g/kg, 0.1 g/kg. 5 g/kg, and 3 g/kg	Post-weaning	From weaning to 42 d of age	LPS	Decreases plasma CD3^+^CD4^−^CD8α^high^ population and TNF-α production	(Lo Verso et al. 2020)
Agrimonia procera, flavonoids and agrimoniin	10 g/kg	Post-weaning	21 days	LPS	Releases more TNF-α in the plasma associated with increasing the expression of DEFB1, GPX2, CXCL1 and IL-8	(Gräber et al. 2018)
Scutellaria baicalensis extracts	1000 mg/kg	Post-weaning	16 days	*E. coli* K88	Reduces IL-1β, TNF-α, IL-6, IgA and IgG production in plasma; decreases the small intestinal TNF-α and IL-1β elevation and inhibits the activation of NF-κB/P38 signaling pathways	(Huang et al. 2019)
*Macleaya cordata* (sanguinarine)	15 g/ton or 50 g/ton	Post-weaning	42 days	None	Reduces the blood concentrations of haptoglobin and serum amyloid A	(Kantas et al. 2015)

LPS, Lipopolysacchar

In piglets challenged with various stressors such as lipopolysaccharides (LPS) (Gräber et al. 2018; Lo Verso et al. 2020; Stelter et al. 2013) or *E. coli* (Xu et al. 2020b; Yang et al. 2019), phytobiotics consistently suppress intestinal inflammation and improve their immune status. Chinese medicinal herbs can reduce diarrhea frequency in weanling pigs by increasing the respiratory burst and *Salmonella*-killing ability of polymorphonuclear cells (Huang et al. 2012). The leukocyte population (*i.e.*, CD3^+^CD4^−^CD8α^high^ T cells) is also altered while TNF-α production is inhibited in piglets given feed additives containing cranberry extract, encapsulated carvacrol, yeast-derived products, and vitamins complex (Lo Verso et al. 2020). Additionally, agrimonia procera increases the release of TNF-α in the plasma of LPS-treated piglets associated with the increasing expression of DEFB1, GPX2, CXCL1 and IL-8 (Gräber et al. 2018). It is suggested that in piglets with stress, phytobiotic supplementations can suppress inflammation through inhibition of the NF-κB/P38 signaling pathways (Cappelli et al. 2021; Huang et al. 2019). It is suggested to be co-opted by the nuclear factor-erythroid 2-related factor-2 (Nrf2), as Nrf2 can be activated by the essential oil, translocated into the nucleus, and prevents the activation of NF-κB (Zou et al. 2016). It is also worth mentioning that in these *in vivo* studies, the immuno-modulatory effects of essential oils have often been associated with changes in gut microbiota, including enriched *Lactobacillus* group and/or a reduction of pathogen loads (Firmino et al. 2021).

Other than the varied results and unclear mechanisms mentioned in probiotics and prebiotics, there are several concerns in using essential oils as feed additives: *i.* potential toxic effects with their lipophilic characteristics, which may impair the liver, GI tract and the reproductive system of animals in high concentrations (Horky et al. 2019); *ii.* possible interactions with other dietary components including sugar, fat and protein, leading to compromised antimicrobial activity and efficacy, which may be resolved by microencapsulation (Lo Verso et al. 2020; Perricone et al. 2015; Wang et al. 2021b; Xu et al. 2020b); *iii.* high inclusion costs in pig production due to their volatility and the requirement of a minimal inhibitory concentration (MIC) for killing bacteria (Lambert et al. 2001; Mariotti et al. 2022). Meanwhile, other phytobiotics including tannins, saponin herbs, and alkaloids also exhibit anti-microbial actions. For instance, tannin extracts can inhibit bacterial growth by iron deprivation, or affect cellular membrane enzymes of microbes; while alkaloids can disrupt bacterial DNA synthesis via topoisomerase inhibition. However, so far, the immuno-modulatory effects of these phytobiotics in swine are mostly explained by their anti-oxidant activities (Girard and BeeG. 2020; Huang et al. 2018). Future studies should therefore focus more on dissecting changes in various immune components of GALT in response to individual phytobiotics to fully unlock their potential as antibiotics alternatives in pig production.

### Others

#### Zinc oxide (ZnO)

Following the ban of in-feed antibiotics in livestock in Europe in 2006, ZnO supplementation has quickly become a popular alternative. In a survey carried out in Spain in 2014, it was shown that more than half of piglets had received ZnO pre-weaning, and the percentage maxed to 73% during the growth stage (Moreno 2014). High levels of ZnO inclusion exhibits anti-bacterial properties, which can protect against post-weaning diarrhea and infections of piglets (Bonetti et al. 2021; Johanns et al. 2019). In challenged piglets, ZnO supplementations modified the gene expressions of SOCS (suppressor of cytokine signaling proteins) involved in inhibiting the JAK-STAT signaling pathway in ileal GALT (Schulte et al. 2016); as well as stimulated the production of IL-1β, IL-6, IL-8, IL-10, and TNF-α in the serum (Guan et al. 2021); whereas in healthy piglets, upregulated gene expressions of ZO-1, IL-10, TGF-β1, and increased SIgA production in the small intestine were reported, thus reducing the incidence of diarrhea (Shen et al. 2014). Meanwhile, Kloubert et al. (2018) and Pei et al. (2019) have demonstrated that ZnO supplementation improved both innate and adaptive immunity of healthy piglets, including the changed activity of natural killer cells (Kloubert et al. 2018; Pei et al. 2019), enhanced IgA and cytokine concentrations (Pei et al. 2019) and increased number of Tregs (Kloubert et al. 2018). Likewise, it is recently shown that piglets exhibited strong T cell reactions in response to ZnO treatments, including the increased population of T-bet^+^, FoxP3^+^, RORγt^+^ and GATA3^+^ T cells in the GALT of piglets (Oh et al. 2021). However, given the suspected environmental pollution, and the fact that ZnO may promote AMR in high dosages, Europe has restricted the use of veterinary drugs containing ZnO in livestock to a maximum level of 150 ppm from 2022 (Bonetti et al. 2021; Ciesinski et al. 2018), suggesting our search of AGPs alternatives must continue.

#### Antimicrobial peptides

Antimicrobial peptides (AMPs) are a diverse class of naturally occurring defense molecules that are produced by many multicellular organisms. They are embodied with active anti-bacterial, anti-fungal, anti-viral, and even anti-cancer properties, and can be used to treat bacterial infections, especially those caused by multidrug-resistant pathogens (Rima et al. 2021). For instance, positively charged AMPs can kill microorganisms by selectively binding to their membranes through electrostatic interactions, disrupting their integrity and/or further affecting their intracellular functions (Zhang and Gallo 2016). In addition, AMPs exhibit the growth-promoting ability and modulate host immunity, making them attractive antibiotic substitutes in swine (Ghosh et al. 2019; Rima et al. 2021; Xu et al. 2020a). It is also well-established that most AMPs are innate and adaptive immune effector molecules that can modulate pro- and anti-inflammatory responses and chemotactic activity (Rima et al. 2021). Dietary supplementation of an AMPs mixture sufficiently improved the cellular immune functions in healthy weaned piglets, reducing splenic cell apoptosis, increasing CD4^+^ T cell populations in blood, and enhancing their proliferation (Ren et al. 2015). While lactoferrin supplementation in newborn piglets significantly increased IgA, IgG and TGF-β1 levels in circulation, enhanced the intestinal integrity and reduced the mortality (Sarkar et al. 2023). Furthermore, AMP addition improved the jejunum barrier integrity, increased the IgM levels and modified the cytokine IL-10, IL-12 and TGF-β concentrations in blood in healthy weaning piglets (Liu et al. 2022b). The immuno-enhancement effect of AMPs on the levels of immunoglobulins in piglets was consolidated by Xu et al. (2020a, 2020b) in a meta-analysis, along with the significantly improved growth performance (Xu et al. 2020a). In addition, some AMPs have been shown to stimulate angiogenesis and chemotaxis and promote leukocyte activation and differentiation (Ghosh et al. 2019; Zhang and Gallo 2016).

## Concluding remarks

The goal of “One Health” is to interconnect human beings, animals, plants, and microorganisms in a hopefully harmonious environment, to achieve optimal health outcomes for all on the earth (Destoumieux-Garzón et al. 2018). During industrialization, the overuse of antibiotics, improved hygiene, and the transformation of the dietary structure have brought about a loss of microbiota compositional and functional diversity and an overall alteration of immunophysiology, finally conceptualizing “The Hygiene Hypothesis” (Gao et al. 2019; Gresse et al. 2017; Pfefferle et al. 2021; Yang et al. 2022). As it is better to prevent than cure, we focus on searching for antibiotic substitutes that improve the immunophysiological state of pigs. We argue that any potential antibiotic alternatives must be safe for animals and the public without encouraging AMR; they should be able to confer health benefits. Preferably they may exhibit specific immuno-modulatory properties and economic feasibility in swine.

Numerous alternatives have been developed to replace antibiotics to improve the health and management of pigs, including probiotics, prebiotics, phytobiotics, organic acids, fermented liquid feeds, enzymes, minerals, proteins/antibodies, AMPs *etc.* Among these, probiotics have made the most progress in research and application in livestock, offering options for animal health improvement. However, as opposed to the broad spectrum of antibiotics, their effects are often strain- or species-specific. Natural plant-derived prebiotics and phytobiotics hold a positive image in the public eye, and some of them are more cost-effective than others. However, whether their immuno-modulatory effects are direct or indirect is still unclear. Antimicrobial peptides behave most like antibiotics, and attracted a lot of attention but are still in their nascent stage of application at large. In conclusion, the data from various studies discussed in this review show progress in developing alternatives to antibiotics in pig production.

## Future perspectives

In search of in-feed supplements to promote performance and health, relationships between immuno-modulation and the physiological status of pigs should be addressed. Especially the intestinal immunophysiology that maintains the host homeostasis through its digestive functioning and balancing between oral tolerance and immune activation through a complex cellular and molecular network. Future studies should focus on documenting the immuno-modulatory effects of antibiotic alternatives in swine. Dissect the porcine immune cell landscape using next-generation sequencing and multi-omics methodology, verify individual regulatory pathways and outcomes of the tested candidate, and evaluate its efficacy. Questions remain as to what is the desired immunity and physiological state? What is the relative importance of gut microbiota? And how to differentiate it? Can a combination strategy of different biologics with immuno-modulatory agents circumvent some limitations and open up new avenues in pig health management? Are there new methods and technologies that can expedite the development of antibiotic alternatives?

## Abbreviations

AGPs: antibiotic growth promoters
AMR: antimicrobial resistance
AMP: antimicrobial peptide
APC: antigen-presenting cell
APRIL: a proliferation-inducing ligand
CFU: colony-forming unit
DC: dendritic cell
FAE: follicle-associated epithelium
FMT: fecal microbiota transplantation
FOS: fructooligosaccharides
HRV: human rotavirus infection
HSPs: heat shock proteins
IETs: intraepithelial T cells
IL: interleukins
ILF: isolated lymphoid follicle
GALT: gut-associated lymphoid tissue
GI: gastrointestinal
LP: lamina propria
LPS: lipopolysaccharides
MAMP: microbe-associated molecular pattern
MIC: minimal inhibitory concentration
MLNs: mesenteric lymph nodes
Nrf2: nuclear factor-erythroid 2-related factor-2
PPs: Peyer’s patches
PPAR-γ: peroxisome proliferator-activated receptor-γ
PRRs: pattern recognition receptors
SCFAs: short-chain fatty acids
SIgA: secretory IgA
SOCS: suppressor of cytokine signaling proteins
TLR: toll-like receptor
Treg: regulatory T cell
ZnO: zinc oxide
